# Subject-specific factors affecting particle residence time distribution of left atrial appendage in atrial fibrillation: A computational model-based study

**DOI:** 10.3389/fcvm.2023.1070498

**Published:** 2023-03-13

**Authors:** Soroosh Sanatkhani, Sotirios Nedios, Prahlad G. Menon, Samir F. Saba, Sandeep K. Jain, William J. Federspiel, Sanjeev G. Shroff

**Affiliations:** ^1^ Department of Bioengineering, University of Pittsburgh, Pittsburgh, PA, United States; ^2^ Department of Electrophysiology, Massachusetts General Hospital, Boston, MA, United States; ^3^ Heart Center, Department of Electrophysiology, University of Leipzig, Leipzig, Germany; ^4^ Cardiovascular Research Institute Maastricht (CARIM), Department of Cardiology, Maastricht University Medical Center, Maastricht, Netherlands; ^5^ Heart and Vascular Institute, UPMC Presbyterian, Pittsburgh, PA, United States

**Keywords:** mean residence time, computational fluid dynamics, confounding variables, pulmonary vein flow, pulsatility, hematocrit, simulation length

## Abstract

**Background:**

Atrial fibrillation (AF) is a prevalent arrhythmia, that causes thrombus formation, ordinarily in the left atrial appendage (LAA). The conventional metric of stroke risk stratification, CHA_2_DS_2_-VASc score, does not account for LAA morphology or hemodynamics. We showed in our previous study that residence time distribution (RTD) of blood-borne particles in the LAA and its associated calculated variables (i.e., mean residence time, *t_m_*, and asymptotic concentration, *C*_∞_) have the potential to improve CHA_2_DS_2_-VASc score. The purpose of this research was to investigate the effects of the following potential confounding factors on LAA *t_m_* and *C*_∞_: (1) pulmonary vein flow waveform pulsatility, (2) non-Newtonian blood rheology and hematocrit level, and (3) length of the simulation.

**Methods:**

Subject-Specific data including left atrial (LA) and LAA cardiac computed tomography, cardiac output (CO), heart rate, and hematocrit level were gathered from 25 AF subjects. We calculated LAA *t_m_* and *C*_∞_ based on series of computational fluid dynamics (CFD) analyses.

**Results:**

Both LAA *t_m_* and *C*_∞_ are significantly affected by the CO, but not by temporal pattern of the inlet flow. Both LAA *t_m_* and *C*_∞_ increase with increasing hematocrit level and both calculated indices are higher for non-Newtonian blood rheology for a given hematocrit level. Further, at least 20,000 s of CFD simulation is needed to calculate LAA *t_m_* and *C*_∞_ values reliably.

**Conclusions:**

Subject-specific LA and LAA geometries, CO, and hematocrit level are essential to quantify the subject-specific proclivity of blood cell tarrying inside LAA in terms of the RTD function.

## 1. Introduction

Atrial Fibrillation (AF), the most common type of arrythmia, was estimated to afflict 33.5 million people globally in 2010 (1). The prevalence of this arrythmia has been estimated to be increased to 15.9 million people in the United States alone by 2050 if the incidence trend continue to rise (2–4). AF patients are clearly at an elevated risk of morbidity and mortality. The most dangerous complication is thromboembolism (TE) for which AF is an independent risk factor. The loss of effective atrial contractile function and sinus rhythm contribute to reduction in cardiac output and leads to flow stasis and thrombus formation, and consecutively raises the risk of cardioembolic events and stroke. AF patients have a 3–5 fold higher risk of stroke and it is estimated that about 15% to 20% of strokes in the US each year can be related to AF (5, 6).

Many of these strokes are caused by thrombi originating in the left atrial appendage (LAA) due to its complex morphology that is conducive to blood stasis: 91% and 50% of thrombi in nonvalvular AF and valvular AF, respectively, are found in the LAA (7–9). Each patient is evaluated for TE risk. Currently, clinical data are the sole factors that are being used to predict stroke and TE risks in AF patients in a clinical setting, with CHA_2_DS_2_-VASc score being the most common metric (10). Efforts have been made to improve the risk stratification for thromboprophylaxis to find the higher risk patients more effectively (11). However, many inconsistencies have been reported among the risk stratification schemes (12).

Several studies have employed computational fluid dynamics (CFD) to analyze the blood flow fields in LA and LAA. In these studies, surrogates of blood flow fields have been studied to associate the dynamics of the blood flow inside the LA and LAA to risk of clot formation. There are several examples of these surrogates, including but not limited to: shear strain rate, wall shear stress (13, 14), oscillatory shear index, time-averaged wall shear stress (15, 16), time-averaged velocity (13, 17–21), particle resident time (22, 23), local relative residence time (16, 24–26), residual virtual contrast agent (13, 18, 19, 27), vortex structure (14, 17–20, 25), flow kinetic energy (25), age stasis (28), and endothelial cell activation potential (ECAP) (16, 29–31). The most accurate approach to simulate clot formation is to include the mechanics of the blood cell (i.e., red blood cells, platelets, etc.) transport into the model, and couple it to the models of thrombus formation and coagulation cascade processes. This approach is associated with substantial computational cost to perform multiscale simulations (32). Qureshi, et al. (21) were able to model thrombogenesis in LA and LAA in a small cohort using a simplified coagulation model. They showed that increased blood stasis in the LAA results in accumulation of thrombin which can lead to thrombus (21). A well-known method to characterize stasis and propensity of blood cells to reside inside the LAA is to calculate the residence time of discrete phase blood borne particles inside the LAA using the Lagrangian approach. However, this approach requires tracking of many individual particles as well as a very fine grid to resolve the flow field with sufficient resolution, making it computationally too expensive. Alternatively, the Eulerian approach can be used to characterize spatial and temporal distributions of blood-borne particle concentration, as opposed to tracking each individual particle. The Eulerian approach, which significantly reduces the computational cost, has been utilized for quantifying indices correlated with thrombus formation (33–36). We have recently reported that blood-borne particle residence time distribution (RTD) and its associated variables (i.e., mean residence time, *t_m_*, and asymptotic concentration, *C*_∞_), calculated using a CFD model of LA and LAA hemodynamics and the Eulerian approach, have the potential to enhance the ability of CHA_2_DS_2_-VASc score to stratify stroke risk in AF subjects (35). Subject-Specific LA and LAA geometries, cardiac output (CO), and heart rate (HR) were used. However, the same temporal pattern of LA inlet flow (i.e., pulmonary vein, PV, flow) was assigned for all AF subjects and simulations were performed for a fixed duration (150 s). In addition, we treated blood as a Newtonian fluid and used a fixed dynamic viscosity value for all AF subjects. It is reasonable to expect that these assumptions may have an impact on the calculated LAA RTD (i.e., values of LAA *t_m_* and *C*_∞_). Accordingly, the purpose of this study was to investigate the effects of the following potential confounding factors on calculated values of LAA *t_m_* and *C*_∞_: (1) PV flow waveform pulsatility (magnitude and temporal pattern), (2) non-Newtonian blood rheology and hematocrit level, and (3) length of the simulation.

## 2. Methods

### 2.1. Data acquisition

All study subjects included in this study were undergoing evaluation and treatment of AF, including medical management and procedural based treatments. Children were excluded from this study. Cardiac-computed tomography (CCT) images were obtained before AF catheter ablation procedure as a part of AF treatment at Heart and Vascular institute (University of Pittsburgh Medical Center; UPMC, Pittsburgh, PA, United States) and Heart Center (University of Leipzig, Leipzig, Germany). Multidetector Helical scanners with 64 and 256 rows were used (Brilliance 64, Philips, Netherlands and Revolution Apex, General Electric Medical System, LLC., Chicago, IL, United States). Electrocardiogram (ECG)-gated acquisition was employed to one beat in cranio-caudal orientation from the aortic arch onto the diaphragm. The acquisition parameters were: 0.6 mm beam collimation, 0.625–1.25 mm thickness, 70–120 kV, 850 mA s, and 20–30 cm field-of-view. Iodinated contrast agent (Ultravist 370, Bayer Vital, Cologne, Germany) was injected (90 ml) during 20 s of end-inspiratory breath holding challenge and a timing bolus-chase injection (20 ml at 5 ml s^−1^). Echocardiography-based measurements of left ventricle short-axis end diastolic and end-systolic diameters were used to calculate stroke volume using the Teichholz formula (37). Cardiac output was calculated as the product of heart rate and stroke volume. Subjects included in this study were part of another study focused on establishing a clinical database of subjects undergoing evaluation and treatment of AF, including medical management and procedural based treatments (i.e., ablation, device-based therapies with pacemakers/defibrillators, and LAA closure devices).

### 2.2. Imaging, segmentation, and computational fluid dynamics

Contrast-enhanced CCT DICOM images of 25 AF subjects with distinctive LAA morphologies were processed to obtain a 3D representation of the LA surface, including the LAA and four pulmonary venous inlets, until the mitral valve plane. The LA-LAA surface geometries were segmented manually. The images were cropped and smoothed using a median filter with a kernel of 5 × 5 × 5 in ParaView (version 5.9.0, Kitware, Inc., Albuquerque, NM, United States). The Marching Cubes method was used to generate an iso-surface representing the LA surface, which included the PV, LA and LAA walls, and the mitral valve plane (excluding the valves themselves). The extracted surface was smoothed out for computational fluid dynamics mesh using Geomagic Studio (version 10, Geomagic, Inc., Research Triangle Park, NC, United States) and ANSYS SpaceClaim (version 2020 R2, ANSYS Inc., Canonsburg, PA, United States) to remove spikes and reduce noise (i.e., simplifying polygons). A detailed flowchart of the LAA segmentation process is provided in Sanatkhani and Menon (38). In short, the size of mesh elements was adjusted based on surface curvature to accurately reflect the topology. As an example, the mesh at the end of the LAA is more detailed than at the center of the LA. The processed geometries were meshed in ANSYS Meshing (version 2020 R2, ANSYS Inc., Canonsburg, PA, United States). The methods and parameters used to mesh the geometries were based on Sanatkhani, et al. (35), with a smaller maximum tetrahedron edge length of 3 mm. Although the total number of mesh elements were typically ∼800,000 tetrahedrons, up to 2,000,000 tetrahedrons were used for subjects with large and complex LAAs.

Blood density was considered *ρ* = 1,060 kg m^−3^ and in case of Newtonian fluid assumption, the dynamic viscosity was considered *μ* = 0.00371 Pa s when studying the effects of pulmonary waveforms as a confounder (Section 3.2.1) and was adjusted according to the hematocrit level when studying the effects of hematocrit and non-Newtonian model as a confounder (Section 3.2.2). The related governing equations have been discretized using spatial and temporal discretization schemes in OpenFOAM (version 8, The OpenFOAM Foundation Ltd, Inc., UK.). Throughout this study walls were assumed to be impermeable, rigid, and with no-slip boundary conditions where pressure gradient is zero. Further, the mitral valve was supposed to be wide open for simplicity and reducing the computational costs. Neumann boundary condition was used at the mitral valve where both gauge pressure and velocity gradient set to zero. Furthermore, the outlet (i.e., mitral valve) was extended to prevent outlet backflow divergence while developing a uniform flow with zero velocity gradient and zero pressure gradient at the outlet. Inlets were set with a Dirichlet boundary condition where a blood velocity inlet profile was given at PV inlets based on PV flow waveform. The PV inlets were cropped to ensure that all subjects had four PV inlets. The flow rate was distributed among the PV inlets based on their cross-sectional area, resulting in uniform and equal velocity inlets for all PV inlets.

More detailed explanation regarding the imaging, segmentation, and CFD methods is presented in Sanatkhani, et al. (35, 38).

### 2.3. Quemada viscosity model

Due to the focus of this study around the stasis region (very low shear strain rate) inside the LAA, it is crucial to take into account the effects of the shear thinning behavior of whole blood. Further, it has been shown that blood viscosity is very sensitive to hematocrit (39).

Using conservation of momentum, the equation of motion (Cauchy's equation of motion) is:

(1) $$\rho \frac{Du_{i}}{Dt}=\frac{\partial \tau_{ij}}{\partial x_{j}}$$

where *D*/*Dt* is material derivative, *t* is time, *x* is coordinate direction, *ρ* is density, *τ* is stress tensor, and *u* is velocity. To include blood viscosity properties in our model we used generalized Newtonian fluid assumption where viscosity depends on the shear rate. Based on this assumption, the constitutive equation for an incompressible fluid using Stokes assumption can be written as follows (40):

(2) $$\tau_{ij}=-\left(\;p+\frac{2}{3}\mu \nabla \cdot u\right)\delta_{ij}+2\mu e_{ij}$$

where *p* is pressure, *δ* is Kronecker delta, *µ* is viscosity, and *e_ij_* is the strain rate tensor^1^. Equation (2) can be substituted into Equation (1) to derive the general form of Navier-Stokes equation. The strain rate tensor in Equation (2) is given by:

(3) $$e_{ij}=\frac{1}{2}\left(\frac{\partial u_{i}}{\partial x_{j}}+\frac{\partial u_{j}}{\partial x_{i}}\right)$$

Due to the small mesh size, especially inside the LAA, we assumed that a single value of shear strain rate will apply in all directions. With the assumption of generalized Newtonian fluid, we calculated the magnitude of strain rate, $\dot{\gamma }$, as follows (39):

(4) $$\dot{\gamma }=\sqrt{2(e_{ij}e_{ij})}$$

Based on the calculated strain rate, $\dot{\gamma }$, at each time-step and each mesh cell the viscosity model was updated to calculate the appropriate apparent viscosity for each cell (32). The Quemada viscosity model (41, 42) has been chosen as a reliable approach to approximate the non-Newtonian properties of blood especially in the LAA where strain rate is low. We employed the Quemada viscosity model in the present study for several reasons: (1) It incorporates blood hematocrit as an explicit parameter, (2) It reproduces the blood non-Newtonian behavior well and matches the performance compared to other available models (43), and (3) It is relatively simple to implement this blood rheological characterization in the CFD code. Based on the Quemada model the blood apparent viscosity, *μ_a_*, can be calculated as:

(5) $$\mu_{a}=\mu_{p}(1-0.5k\mathrm{Hct}{)}^{-2}$$

where *μ_p _*= 0.00123 Pa s is plasma viscosity and Hct is hematocrit level. Coefficient *k* and its other related coefficients are calculated using the relations in Table 1.

**Table 1:** Quemada viscosity model coefficients.

$k=\;\frac{k_{0}+k_{\infty }\sqrt{\dot{\gamma }//{\dot{\gamma }}_{c}}}{1+\sqrt{\dot{\gamma }//{\dot{\gamma }}_{c}}}$
$k_{0}=\exp(3.874\;-\;10.41\;\mathrm{Hct}\;+\;13.8\;\mathrm{Hc}t^{2}\;-6.738\;\mathrm{Hc}t^{3})$
$k_{\infty }=\exp(1.3435\;-\;2.803\;\mathrm{Hct}\;+\;2.711\;\mathrm{Hc}t^{2}\;-\;0.6479\;\mathrm{Hc}t^{3})$
${\dot{\gamma }}_{c}=\exp(-6.1508\;+\;27.923\;\mathrm{Hct}\;-\;25.6\;\mathrm{Hc}t^{2}\;+\;3.697\;\mathrm{Hc}t^{3})$

Hct: hematocrit; $\dot{\gamma }$: shear strain rate; *k*: intrinsic viscosity; ${\dot{\gamma }}_{c}$, *k*_0_, *k*_∞_: Quemada coefficients.

### 2.4. OpenFOAM solvers

Previous studies have concluded that laminar assumption is adequate in context of flow modelling in LA (16, 44). Therefore, we solved the governing equations using a laminar solver developed from nonNewtonianIcoFoam solver in OpenFOAM by implementing the Quemada viscosity model into the nonNewtonianIcoFoam solver. We modified the ScalarTransportFoam solver for implementing the tracer transport simulations and conducted the tracer transport-related simulations only after a steady state flow was reached (after 25 cycles).

We used the asymptotic tracer concentration inside LAA (35) as our convergence criteria to choose the time step for our simulations. A time-step study was carried out in which independence of solutions to time-step = 500 µs was established. The first-order implicit and second-order least-square methods were used for time and pressure (as well as velocity gradient) discretization, respectively. Divergence terms and convection terms were discretized using first-order and second order upwind schemes, respectively. Tolerances for velocity, pressure, velocity, and concentration were set to be 10^−8^ m/s, 10^−7^ Pa, and 10^−8^, respectively. For these simulations, 24 threads of dual 12 core Intel Xeon Gold 6126 CPU with 2.6 GHz clock speed and minimum of 8 GB of RAM were used at the University of Pittsburgh Computing Research Center. The average execution time for each case in a steady flow using non-Newtonian model was ∼7 days to simulate 20,000 s of tracer concentration advection through LA/LAA. The codes and instructions regarding the solvers developed for this study have been made available *via* the project repository (https://github.com/sorooshsanatkhani/LAA-AF-Stroke).

### 2.5. LAA residence time distribution of blood-borne particles and associated indices

Previous studies have demonstrated that majority of thrombi in AF originate from the LAA. As a result, the focus of this study was on the LAA, rather than other locations (7–9). LAA RTD of blood-borne particles and associated indices (LAA *t_m_*, and *C*_∞_) were calculated to quantify the propensity of blood-borne particles to reside inside the LAA. The details regarding these calculations, including the graphical representation for the CFD simulations, are presented in (35). In short: tracer transport-related simulations were performed using fluid dynamic analysis to simulate the advection of a tracer through the LAA. The tracer concentration inside the LAA was recorded as *C*(*t*) and fitted to a triple exponential model that included an asymptotic term, *C*_∞_. The residence time distribution (RTD) function was used to quantify the dynamics of tracer clearance from the LAA, with the unit per second representing the normalized outflow of tracer material from the LAA at time *t*. Two measures of the propensity of particles to remain within the LAA were calculated: mean residence time (*t_m_*), which is the first moment of the RTD function, and *C*_∞_ [*C*_∞_ = *C*(*t* → ∞)].

### 2.6. Statistical analysis

Data for continuous variables are presented as mean ± standard deviation. For parameters in linear regression, mean ± standard error of the estimates is reported. Rank correlations between variables were calculated by Spearman rank correlation. Statistical significance for all comparisons was taken to be *P* < 0.05. A multiple linear regression analysis was conducted to identify the effects of 3 independent variables (i.e., CO and 2 PV flow waveform pulsatility indices, Table 2) on LAA *t_m_* or LAA *C*_∞_:

(6) tmorC∞=α+βCO∗CO+βSys∗SysP+βRev∗RevP+∑i=124γiDi

where, *α* is the intercept and *β*'s are the coefficients of the independent predictor variables in the regression model. The last term in Equation (6) is included to account for the inter-subject variability of the intercept, where a set of 24 dummy variables are defined using effects coding (45):

(7) $$D_{i}(i=1:24)=\{\begin{array}{cc} 1,\; & \mathrm{Observation}\;\mathrm{is}\;\mathrm{from}\;\mathrm{Subject}\;i\;\\ -1,\; & \mathrm{Observation}\;\mathrm{is}\;\mathrm{from}\;\mathrm{Subject}\;25\;\\ 0,\; & \mathrm{Otherwise}\; \end{array}$$

**Table 2:** Pulmonary vein blood flow waveform pulsatility indices.

Waveform Pulsatility Indices	Normal Pulsatile Waveform	AF Pulsatile Waveform	No Pulsatile Waveform	Cardiac Output (L min^−1^)
1 $\mathrm{Normalized}\;\mathrm{Systolic}\;\mathrm{Peak}(\mathrm{Sy}s_{P})=\frac{\mathrm{Systolic}\;\mathrm{Peak}}{\mathrm{Cardiac}\;\mathrm{Output}}$	2.25 2.18 2.27	1.55 1.25 1.54	1 1 1	3.3 4.4 5.5
2 NormalizedReversalPeak(Revp)=|ReversalPeak|CardiacOutput	0.64 0.91 0.91	0.61 0.18 0.21	1 1 1	3.3 4.4 5.5

**Figure 1:**
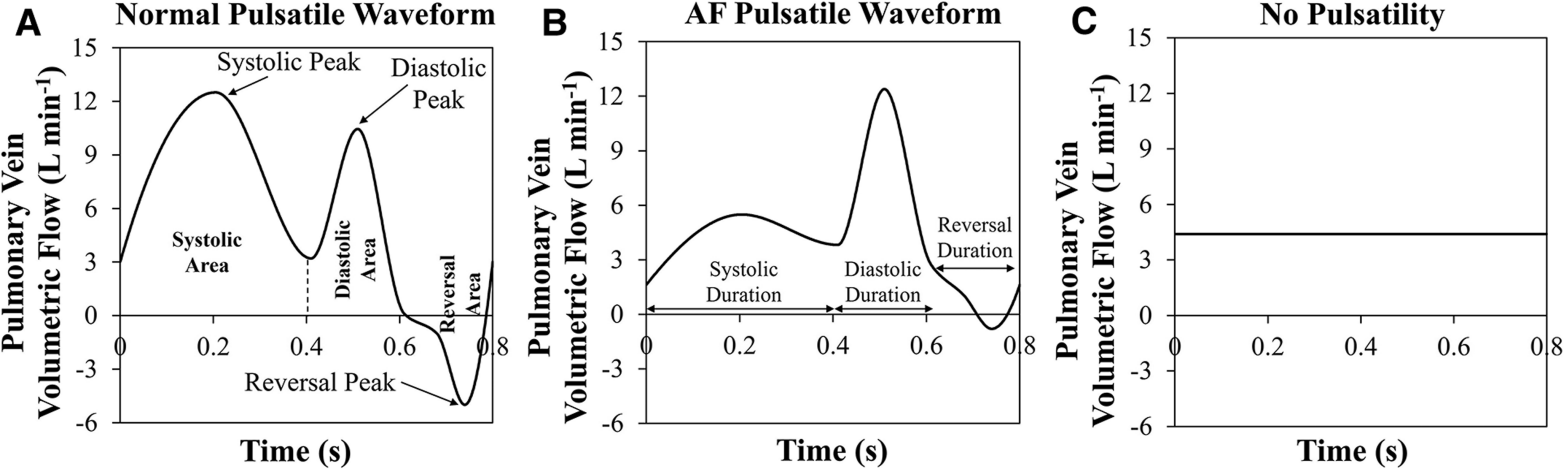
**Three pulmonary vein flow waveform types**. (**A**) Normal pulsatile pulmonary vein (PV) flow waveform. Systolic, diastolic, and reversal areas in during one cardiac cycle are shown. Further, the peak of each period is pointed out. (**B**) Pulsatile PV flow waveform that is seen in a typical atrial fibrillation patient. Systolic, diastolic, and reversal durations are marked. (**C**) PV flow waveform with no pulsatility.

Definitions of pulmonary vein (PV) flow waveform pulsatility indices are presented. Further, their value corresponding to each waveform type [pulsatile waveform seen in a typical normal subject, pulsatile waveform seen in a typical atrial fibrillation (AF) patient, and steady with no pulsatility; Figures 1A–C] and cardiac output are shown. Systolic peak and reversal peak are shown in Figure 1A.

The design matrix for the dummy variables, *D_i_*, is given in Equation (7).

The effects of hematocrit (3 levels, 27.4%, 45.5%, and 60.4%), blood rheology model (2 levels, Newtonian and non-Newtonian), and their interaction on LAA *t_m_* or LAA *C*_∞_, was tested by a multiple linear regression model:

(8) $$t_{m}\;\mathrm{or}\;C_{\infty }=\alpha +\beta_{\mathrm{Hct}}\;*\;\mathrm{Hct}+\beta_{N}\;*\;D_{N}+\beta_{\mathrm{HD}}\;*\;\mathrm{Hct}\;*\;D_{N}+\sum_{i=1}^{24}\gamma_{i}D_{i}$$

where, *α* is the intercept and *β*'s are the coefficients of the independent predictor variables, *D_N_* is the dummy variable to account for blood rheology model (*D_N_* = 1, if non-Newtonian, *D_N_* = 0, if Newtonian) and *D_i_*'s are the dummy variables to account for the inter-subject variability in the intercept value as before [Equation (7)]. A single CO value (4.4 L min^−1^) with steady PV flow (i.e., no pulsatility) was used in the simulations for this model.

Regression parameter estimates are presented as mean ± standard error. Statistical analyses in this study were carried out in the MATLAB® (version R2022b, MathWorks, Inc., Natick, MA, United States). Additional details about the statistical analysis can be found in the Supplement.

### 2.7. Confounding factors

As discussed above, there are several confounding factors that can affect regarding the CFD-based modeling of hemodynamics and particle transport and consequently, the calculation of LAA residence time. In this section we present the sets of simulation that we used to examine the following confounding factors: (1) PV flow waveform pulsatility (magnitude and temporal pattern), (2) non-Newtonian blood rheology and hematocrit level, and (3) length of the simulation.

#### 2.7.1. Simulation set 1: pulmonary vein flow waveform

Subject-specific 3D geometry can be obtained readily, however, it is not easy to measure all PV inlet blood flow waveforms *in vivo.* A study to investigate the effects of inlet blood flow waveform pulsatility (magnitude and temporal pattern) on LAA residence time is needed to examine whether the nature of the inlet flow (steady vs. pulsatile) affects LAA residence time. Accordingly, various PV blood flow waveforms were generated by modifying the template waveforms (Normal Pulsatile: Figure 1A; AF Pulsatile: Figure 1B; No Pulsatility: Figure 1C).

In our cohort of 25 subjects, each subject was simulated using 9 settings of PV inlet blood flow pulsatility (resulting in a total of 225 observations): 3 levels of mean PV blood flow (i.e., CO = 3.3, 4.4, and 5.5 L min^−1^) and 3 types of PV flow waveform [pulsatile waveform seen in a typical normal subject, pulsatile waveform seen in a typical AF subject, and no pulsatility (steady); Figures 1A–C] for each of the three levels of CO. The mean residence time of blood-borne particles in LAA, LAA *t_m_*, and asymptotic concentration inside LAA, LAA *C*_∞_, were quantified in each simulation.

To investigate the effects of pulsatility of PV blood flow waveforms, we characterized PV blood flow waveform pulsatility in terms of two indices (Table 2). Multiple linear regression analysis was used to identify the effects of CO and 2 PV flow waveform pulsatility indices on LAA *t_m_* or LAA *C*_∞_.

#### 2.7.2. Simulation set 2: non-newtonian blood rheology and hematocrit level

**Figure 2:**
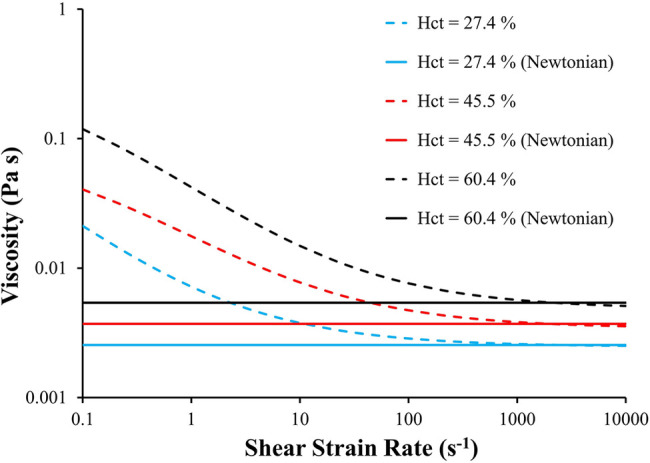
**Blood viscosity as a function of shear strain rate and hematocrit using Quemada viscosity model and Newtonian fluid model**. The equivalent Newtonian viscosity of each hematocrit level was calculated based on the corresponding viscosity calculated using Quemada model at $\dot{\gamma }=2,000\;s^{-1}$. Hct: hematocrit.

We used our cohort of 25 subjects to investigate the effects of hematocrit level and non-Newtonian behavior of blood on the calculated indices (LAA *t_m_* and LAA *C*_∞_). The non-Newtonian behavior of blood was simulated for 3 different hematocrit levels (Hct = 27.4%, 45.5%, and 60.4%) using the Quemada viscosity model. Further, the equivalent Newtonian viscosity of each hematocrit level was calculated based on Figure 2 (*μ* = 2.5 × 10^−3^, 3.7 × 10^−3^, and 5.4 × 10^−3^ Pa s for Hct = 27.4%, 45.5%, and 60.4%, respectively). Six CFD-based simulations were conducted for each subject (resulting in a total of 150 observations): non-Newtonian and Newtonian behavior of blood for each of the 3 levels of hematocrit. A pulmonary vein flow waveform with no pulsatility with cardiac output of 4.4 L min^−1^ was used in these simulations. Multiple linear regression analysis was used to identify the effects of Hct, blood rheology model and their interactions on LAA *t_m_* or LAA *C*_∞_.

#### 2.7.3. Simulation set 3: length of simulation

In theory, one needs to continue the CFD-based simulation of tracer transport to infinite time for calculating the mean residence time (46). Clearly, this is not possible. Therefore, simulations must be truncated at some point in time. LAA *t_m_* and LAA *C*_∞_ values are calculated based on these truncated data and an assumed decay function. Based on our study, the temporal pattern of the LAA tracer concentration decay following an impulse injection of tracer is complex—it is certainly not a single exponential decay. We chose a triple exponential decay function (capable of fitting to a period of fast tracer washout at the beginning of simulation, moderate washout rate in the middle, and slow washout rate at the end of the simulation) as a compromise between over fitting and accuracy. It is important to know what minimum length of simulation is necessary for a reliable calculation of the mean residence time. We calculated LAA *t_m_* and LAA *C*_∞_ for various simulation times over the range 625 s to 30,000 s.

## 3. Results

### 3.1. Study subject characteristics

A total of 25 subjects (15 males) with symptomatic AF (22 paroxysmal, 3 persistent) were studied. The average age, heart rate, cardiac output, and hematocrit level were 61 ± 11 years (range: 33–78 years), 64.1 bpm (range: 44–84 bpm), 3.8 L min^−1^ (1.9–6.8 L min^−1^), and 41.5% (35%–49%). The average CHA_2_DS_2_-VASc score was 1.9 ± 1.1 (range: 0 to 4).

### 3.2. Effects of confounding factors

In this section we present the results of our studies carried out to examine the effects of the following confounding factors: (1) PV flow waveform pulsatility (magnitude and temporal pattern), (2) non-Newtonian blood rheology and hematocrit level, and (3) length of the simulation.

#### 3.2.1. Pulmonary vein flow waveform

**Figure 3:**
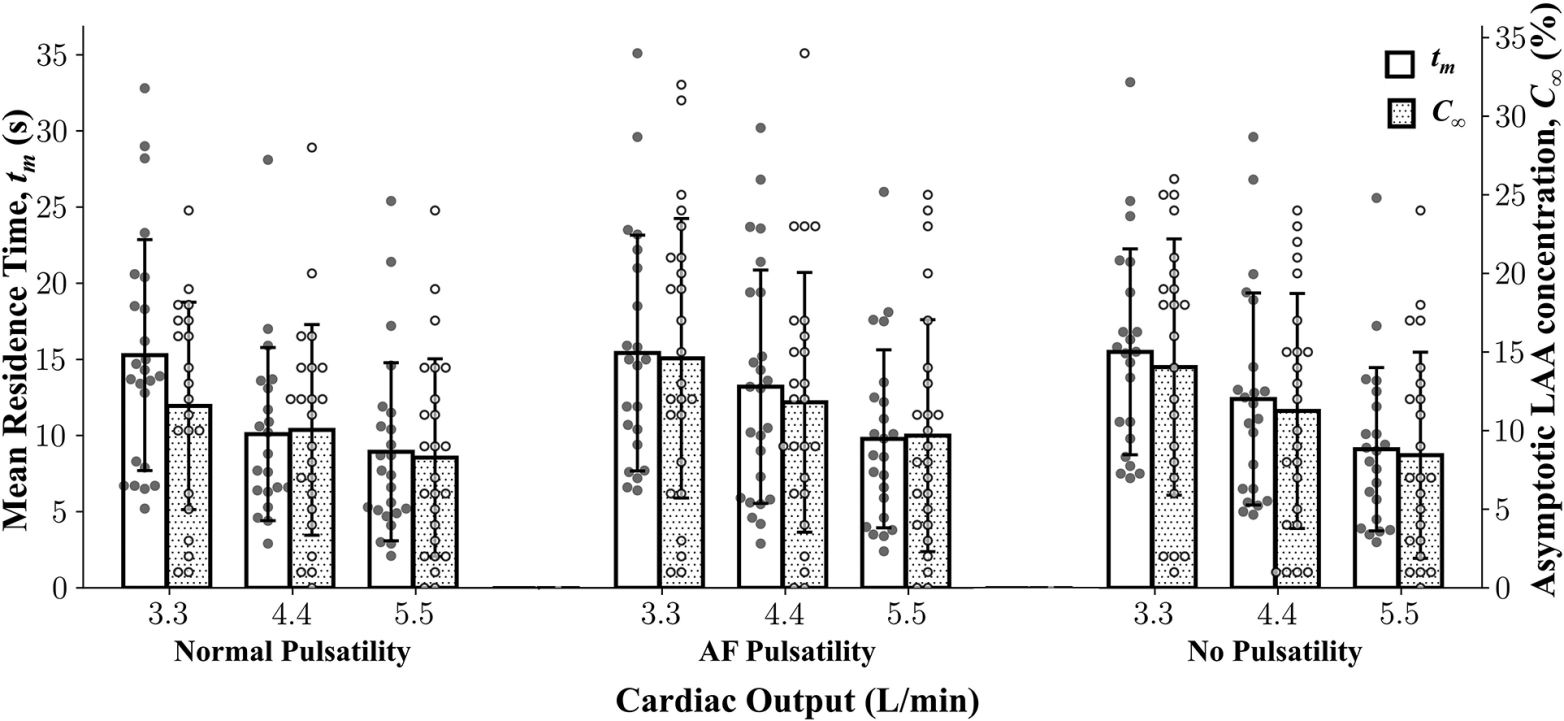
**Three pulmonary vein flow waveform types and their relationship with the hemodynamic indices**. Mean residence time and asymptotic concentration in left atrial appendage corresponding to different PV flow waveforms and cardiac outputs for a cohort of 25 patients. Data: Mean ± SD.

Multiple linear regression analysis showed that only CO was a significant independent predictor variable (i.e., only *β*_CO_ in Equation (6) was significantly different from zero, *P* < 0.0001); none of the coefficients associated with indices of PV waveform pulsatility (i.e., coefficients *β*_Sys_ and *β*_Rev_) were significantly different from zero. This observation implies that both LAA *t_m_* and LAA *C*_∞_ decreased significantly as CO was increased, regardless of PV waveform type (Figure 3). Based on this study, an increase of 1 L min^−1^ in CO decreases the LAA *t_m_* by 2.43 s (±0.20 s; adj-*R*^2^ = 0.87; *P* < 0.0001) and *C*_∞_ by 2.09% (±0.19 s; adj-*R*^2^ = 0.89; *P* < 0.0001).

#### 3.2.2. Non-Newtonian blood rheology and hematocrit level

**Figure 4:**
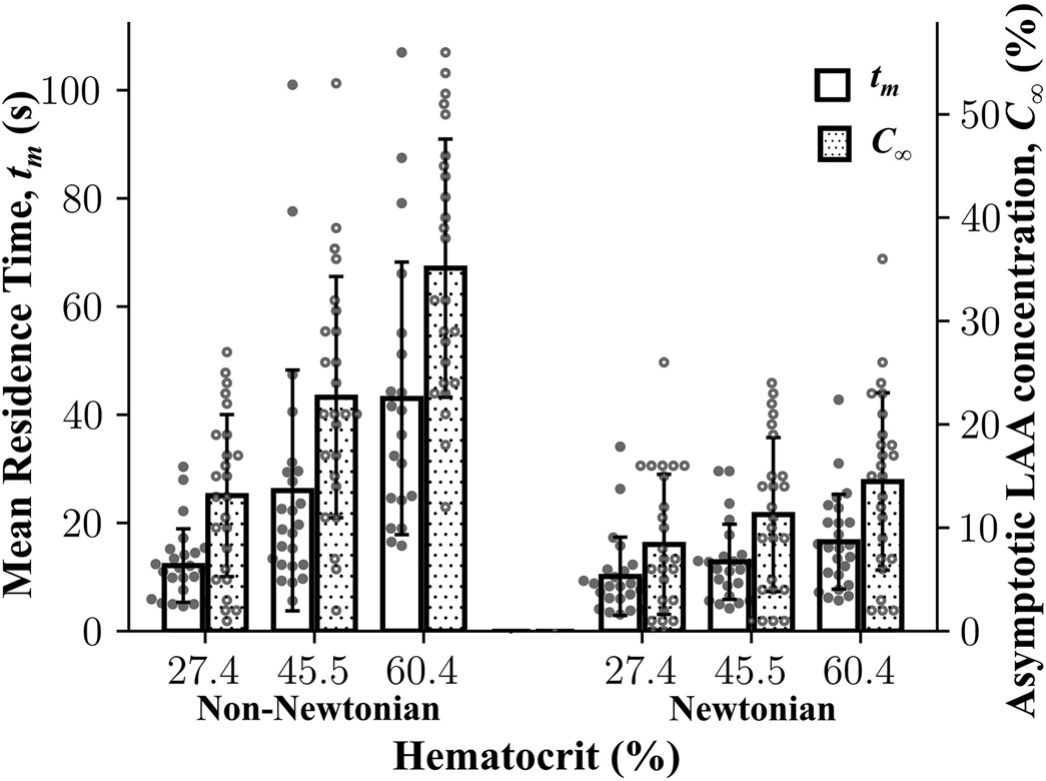
**Mean residence time and asymptotic concentration inside left atrial appendage as a function of hematocrit using Newtonian and non-Newtonian models.** Left atrial appendage mean residence time, LAA *t_m_*, LAA asymptotic concentration, *C*_∞_, increased as a function of cardiac output. Data: Mean ± SD.

The results of multiple linear regression analysis showed that both LAA *t_m_* and LAA *C*_∞_ are significantly affected by Hct, choice of blood rheology, and the interaction between the Hct and blood rheology model (*P* < 0.0001). Both LAA *t_m_* and LAA *C*_∞_ values for a given hematocrit level were significantly lower for the Newtonian model as compared the values for the non-Newtonian model (Figure 4). In both Newtonian and non-Newtonian models, both LAA *t_m_* and *C*_∞_ increased with increasing hematocrit level (Figure 4). The multiple linear regression model was used to relate LAA *t_m_* or LAA *C*_∞_ to hematocrit level using the non-Newtonian fluid characterization in simulations (Quemada viscosity model), respectively. Hematocrit level was found to be a significant independent variable as expected for both LAA *t_m_* (*β*_Hct_ = 0.65 ± 0.07; adj-*R*^2^ = 0.85; *P* < 0.0001) and LAA *C*_∞_ (*β*_Hct_ = 0.65 ± 0.05; adj-*R*^2^ = 0.83; *P* < 0.0001).

To examine whether the fluid characterization (Newtonian vs. non-Newtonian) affects the rank ordering of subjects, we performed the Spearman rank correlation analysis of results obtained using the Newtonian model and the non-Newtonian model (i.e., Quemada model). Based on 150 simulations [75 Newtonian (25 subjects × 3 hematocrit levels) and 75 non-Newtonian], LAA *t_m_* and *C*_∞_ from the non-Newtonian model and the Newtonian model were highly correlated (*ρ* = 0.71, *P* < 0.0001 for LAA *t_m_* and *ρ* = 0.82, *P* < 0.0001 for LAA *C*_∞_).

#### 3.2.3. Length of simulation

**Figure 5:**
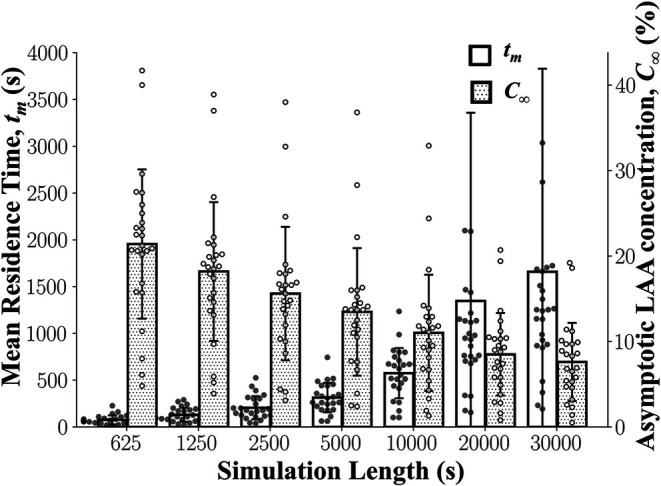
**Left atrial appendage mean residence time, LAA *t_m_*, and asymptotic concentration, *C*_∞_ as a function of simulation length**. LAA *t_m_* and *C*_∞_ did not reach a steady state even after 30,000 s of simulation. Data: Mean ± SD.

It was expected that the calculated LAA *t_m_* and *C*_∞_ values would reach an asymptotic steady state by the end of the 30,000 s simulation. The mean LAA *t_m_* increased and the mean LAA *C*_∞_ decreased as a function of the simulation time (Figure 5). Although some individual subjects reached steady-state after 30,000 s of simulation, it does not appear that the mean LAA *t_m_* and LAA *C*_∞_ for the cohort of 25 subjects reach steady-state values (Figure 5).

**Figure 6:**
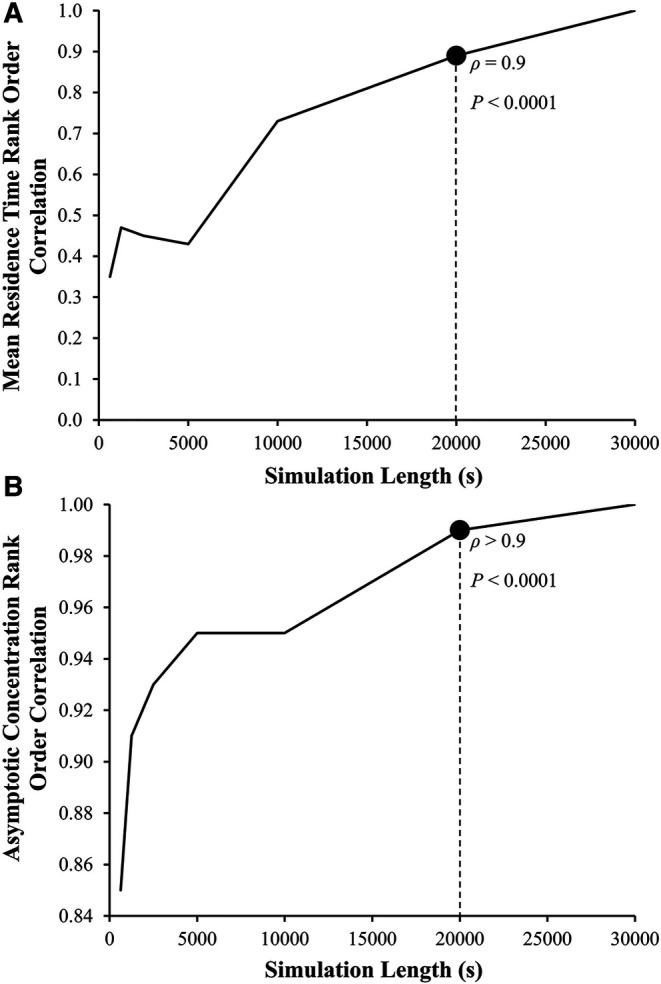
**Left atrial appendage mean residence time, LAA *t_m_*, and asymptotic concentration, *C*_∞_ rank order correlation coefficient as a function of the length of simulation.** The Spearman rank order correlation coefficient, *ρ*, between the LAA *t_m_* and *C*_∞_ for the reference group using 30,000 s of simulation (*ρ* = 1, by definition) and LAA *t_m_* and *C*_∞_ calculated using smaller simulation lengths.

Although reaching a steady state is ideal, the consistency of the rank ordering of subjects is more important. Spearman rank order correlation analyses between LAA *t_m_* and LAA *C*_∞_ values calculated using 30,000 s simulation and results based on shorter simulation lengths were performed. Based on these results, 20,000 s found to be a sufficient length to calculate LAA *t_m_* (*ρ* = 0.9, *P* < 0.0001; Figure 6A) and LAA *C*_∞_ (*ρ* > 0.9, *P* < 0.0001; Figure 6B).

## 4. Discussion

Tarrying of blood cells inside the LAA could lead in an increased risk of thrombus formation and, consequently, stroke. We have recently quantified the proclivity of blood cell staying within the LAA in terms of the RTD function, *E*(*t*), and related calculated variables: mean residence time of blood-borne particles in LAA, *t_m_*, and asymptotic concentration remaining inside LAA, *C*_∞_ (35). Even though it is important for these calculations to be subject-specific, only subject-specific LA and LAA morphologies were used in the previous study. The present study explored the effects of additional subject-specific variables [pulmonary vein (PV) flow waveform pulsatility, cardiac output, and hematocrit] and certain CFD model-related assumptions (Newtonian blood rheology, length of the CFD simulation) on the calculation of LAA RTD function and associated calculated variables (LAA *t_m_* and *C*_∞_). The key observations of the present study are as follows: (1) LAA *t_m_* and *C*_∞_ values are significantly affected by the mean value (cardiac output, but not the temporal pattern) of the PV inlet flow and hematocrit; (2) Although non-Newtonian blood rheology significantly increased both LAA *t_m_* and *C*_∞_, the rank ordering of LAA *t_m_* and *C*_∞_ were similar for Newtonian and non-Newtonian formulations; and (3) The length of CFD simulation should be at least 20,000 s for reliable calculations of LAA *t_m_* and *C*_∞_.

Several indices exist that relate blood flow patterns in LA and LAA to the probability of clot formation. These indices are directly calculated from flow the velocity field (e.g., wall shear stress, time-averaged wall shear stress, oscillatory shear index, time-averaged velocity, vortex structure, flow kinetic energy, and ECAP). In contrast, LAA RTD incorporates the transport of blood-borne particles, and it, by definition, quantifies the propensity of blood cell lingering within the LAA. Although the velocity field-based indices require only a short simulation time, we believe that LAA RTD has the capability to better simulate the transport and lingering of blood cells in LAA.

It has been suggested that the PV flow pattern seen in AF subjects, with diminished systolic flow and end-diastolic flow reversal (17), is associated with hemodynamic indices that predict higher chance of thrombus formation compared to that for the normal PV flow pattern (13). Several studies have shown that the flow pattern within in LA and LAA and LA-LAA wall contraction pattern in AF are the determinants of the thrombus formation (14, 17, 19). However, we showed in this study that PV flow waveform pulsatility does not affect the LAA RTD (i.e., representative of risk of thrombus formation in LAA), an observation that is consistent with the findings of Dueñas-Pamplona, et al. (44), suggesting that LA-LAA wall contraction pattern is more important than PV flow temporal pattern. The LAA blood stasis risk, as quantified by LAA *t_m_* and *C*_∞_, was significantly affected by the mean value of inlet flow (i.e., cardiac output), Therefore, the subject-specific LAA blood stasis risk can be reliably estimated using subject-specific LA and LAA 3D geometries and subject-specific cardiac output, without any need for subject-specific PV blood flow waveform.

The assumption that blood flow inside the left atrium (LA) can be modeled as a Newtonian fluid is considered reasonable due to the high strain rates present in the LA cavity, which cause blood to behave like a Newtonian fluid (13, 14, 16, 20). However, due to the existence of stasis regions inside the LAA and associated low shear strain rate, non-Newtonian blood rheology might be important in calculating LAA *t_m_* and *C*_∞_. We observed that both LAA *t_m_* and *C*_∞_ were affected significantly by hematocrit level and blood rheology (Newtonian vs. non-Newtonian): both LAA *t_m_* and *C*_∞_ values were higher for the non-Newtonian formulation.

Gonzalo, et al. (47) have investigated blood rheology effects on CFD estimations of LAA blood stasis, including LA-LAA residence time. They used the Carreau–Yasuda rheology model parameters to mimic Hct = 37% and 55%. In contrast, we chose the Quemada model because it allows us to explicitly adjust the Hct values. However, both models have been demonstrated to perform well (43). Further, they employed a modified rheology model wherein non-Newtonian effects are activated based on the local residence time. Gonzalo, et al. (47) calculated residence time by solving a scalar advection transport equation where the source term is 1, resulting in an increasing age of fluid over time (48). The mean residence time in a specific region can then be calculated by averaging the age of fluid at each grid point over a period of time. In contrast, the present study follows the concept of mean residence time as described in Fogler (49), which involves solving a scalar advection transport equation with a source term of zero and an initial condition where the region of interest has a scalar (i.e., tracer) concentration of 1. The mean residence time is then calculated based on the concentration of the tracer inside the region as a function of time, as described in more detail in Sanatkhani, et al. (35). Although the methods used to investigate blood rheology effects differ between the Gonzalo, et al. study (47) and the present study, both studies are aiming to identify thrombus-promoting flow patterns and the results of both studies are similar: higher Hct values are associated with higher residence time and there is a greater effect of Hct on residence time at higher Hct values. Researchers can choose between these two methods for modeling blood rheology and calculating mean residence time depending on their specific research goals and the availability of required input parameters.

The choice of blood rheology model did not affect LAA *t_m_* and *C*_∞_ rank ordering among the study subjects. Therefore, one might choose to quantify the LAA *t_m_* and *C*_∞_ in a study cohort using Newtonian fluid model with a fixed value of viscosity corresponding to the subject-specific hematocrit level. However, the incremental computational cost of using a non-Newtonian blood rheology model (i.e., Quemada model) was negligible. Therefore, we recommend that the non-Newtonian blood rheology model be used in all future CFD simulations.

It is important to note that *t_m_* will continue to rise if certain amount of tracer is stuck in the LAA (never gets washed out). This can be readily seen from the definition of *t_m_* (35, 47). On the practical level, estimated *t_m_* and *C*_∞_ will be used to rank order the thrombogenic risk. Our results indicate (Figure 6) that the rank ordering at 20,000 s is more than 90% similar to the rank ordering for 30,000 s. Therefore, it is reasonable to conclude that 20,000 s is a sufficient simulation time. Despite this, the CFD simulation for a single subject still requires a significant amount of computational time (∼7 days using 24 threads of dual 12 core Intel Xeon Gold 6126 CPU with 2.6 GHz clock speed and minimum of 8 GB of RAM). Additional enhancements of the CFD model, such as one-way and two-way fluid-wall interactions and multiscale analysis of biochemical coagulation cascade, will further increase the computational cost. A new method to reconstruct RTD, introduced by Sierra-Pallares, et al. (48), might be able to reduce the computational cost of LAA *t_m_* and *C*_∞_; however, its applicability and accuracy has not been tested using LA-LAA geometries. In recent studies, deep neural network has been implemented to predict CFD simulation results in LA-LAA geometries (29). Although this approach is expected to decrease the computational cost significantly, many CFD simulations are still needed to develop the ground truth for LAA *t_m_* and *C*_∞_ (and any other indices developed in the future) that is necessary for training the deep neural network.

Our data indicate that mean residence time, *t_m_*, and asymptotic concentration, *C*_∞_, are correlated and therefore, they may be used interchangeably. However, if the tracer washes out completely after a certain time, *C*_∞_ will be zero and therefore, *t_m_* is the only index that can be used to discriminate between subjects. We believe that both *t_m_* and *C*_∞_ should be reported to provide a comprehensive understanding of the residence time distribution.

**Figure 7:**
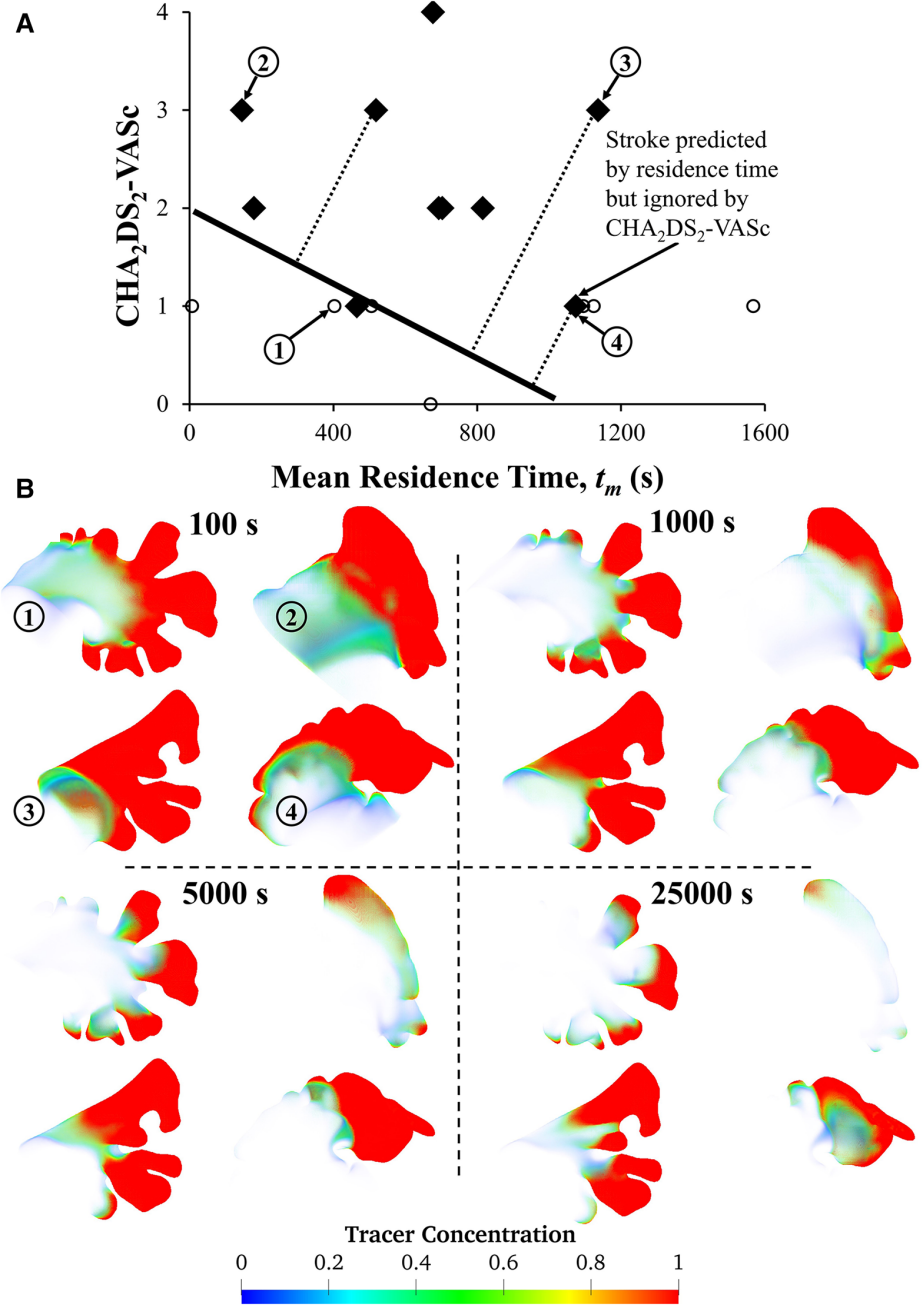
**Relationship between LAA *t_m_* and CHA_2_DS_2_-VASc score and visual representation of tracer washout in the LAA of four subjects.** (**A**) The plot of CHA_2_DS_2_-VASc vs. *t_m_* reveals that a patient with a stroke (marked with diamond symbols) could potentially be overlooked if LA hemodynamics are not considered, as subject #4's tm values indicate a high risk of stroke. Subject #4 has a history of stroke, which is not reflected in their CHA_2_DS_2_-VASc score. However, *t_m_* values may be able to predict the risk of stroke. To evaluate the accuracy of CHA_2_DS_2_-VASc in predicting stroke, data points corresponding to previous strokes were excluded. Only 17 subjects are shown in this figure because complete physiological/clinical data were not available for the remaining 8 subjects. (**B**) Contours of tracer concentration at selected times show the tracer washout in each subject from most of the LAA, with the exception of the tip. Among these four subjects, Subject #2 had the simplest morphology, while Subjects #3 and #4 had more complex morphologies with multiple lobes, long LAA, and a sharp bend.

Finally, we performed a preliminary analysis to explore whether quantifying mean residence time helps stratify stroke risk. The mean residence time was calculated as a function of subject-specific LA-LAA morphology, CO, and Hct. The plot of mean residence time against CHA_2_DS_2_-VASc score (Figure 7A) illustrates that both CHA_2_DS_2_-VASc score and mean residence time may be helpful in stratifying patients. The patient at the bottom right of the figure (Subject #4) has a high stroke risk according to the mean residence time, *t_m_*, but this risk may be overlooked if the focus is only on CHA_2_DS_2_-VASc score. In contrast, the patient in the top left of the figure (Subject #2) has a low residence time but a high CHA_2_DS_2_-VASc score, demonstrating that residence time alone is not sufficient. A discriminative line can be envisioned in the figure to suggest the possibility of using mean residence time along with CHA_2_DS_2_-VASc score to stratify stroke risk in future studies. Data from four subjects are shown to illustrate the variability of the tracer washout among these subjects (Figure 7B). The morphology of the LAA seems to have a direct impact on *t_m_*. As shown, Subject #3 had multiple dominant lobes and Subject #4 had a long LAA with a sharp bend. These complex LAA shapes contributed to the relatively high tracer concentration in the LAA of Subjects #3 and #4 even after 25,000 s. However, the visual complexity of the LAA does not always dictate its residence time. For example, Subject #1 appears to have a complex shape, but due to its high cardiac output, the calculated residence time was not high. In a future study, we will compare the simulation results with thrombogenic events (in the context of developing a prediction algorithm). However, that was not the goal of this study. Comparing thrombogenic events with simulation results for the purpose of risk assessment requires a larger cohort with a sufficient number of thrombogenic events to achieve statistical significance. This is our goal in our next study, where we will collect longitudinal data from a much larger patient cohort. The primary aim of this study was to investigate the effects of some subject-specific variables on the calculation of LAA RTD function and associated calculated variables (LAA *t_m_* and *C*_∞_), so that we can use the “optimized” approach for patient-specific CFD-based modeling in future studies.

## 5. Limitations

Although we examined the effects of some subject-specific and other confounding variables on the calculation of LAA *t_m_* and *C*_∞_, there are additional considerations that merit evaluations. The contractility pattern of the LA-LAA wall during atrial fibrillation (AF) has been shown to increase the risk of thrombus formation as predicted by fluid dynamics indices. Rigid wall simulations are insufficient in modeling these effects (14, 16–18, 20, 21, 25, 27, 44). In this study, we accepted the rigid wall assumption as a limitation for two reasons: (1) A 4D data set (such as CT or MRI) is needed to impose LA-LAA wall motion as a boundary condition for more sophisticated fluid-structure interaction models that require LA-LAA passive and active wall mechanical properties (17, 20). These data were not available for this study. Additionally, using population average wall motion patterns from literature (which implies using the same temporal pattern of movement for all subjects) is unlikely to alter the ranking of subjects (more on this in point #2). (2) In our follow-up study, which aims to assess stroke risk, we value the ranking of calculated mean residence time among subjects. Studies have shown that rigid wall assumptions may overestimate thrombogenesis risk, as expected. However, there is no conclusion that this assumption would alter the ranking of calculated variables. While there are studies in the literature that have included wall motion in their simulations (14, 17, 19, 44), they have only included a small number of subjects, which is not suitable for risk assessment. We acknowledge these limitations in the present study and future parametric studies that examine the effects of LA-LAA wall properties and contraction patterns on LAA *t_m_* and *C*_∞_ in larger cohorts are needed.

For simplicity, we assumed the mitral valve to be wide open in the CFD simulations. Further, both gauge pressure and velocity gradient were set to zero. It is possible that a more realistic (physiologic) outlet boundary condition will affect the calculation of LAA *t_m_* and *C*_∞_. It is postulated that the presence of mitral regurgitation (MR) modifies the stroke risk in AF subjects; but this issue is still controversial (49). Incorporating the models of mitral valve and left ventricular diastolic behavior will enable us to study the effects of the outlet boundary conditions (17). Further, it has been shown that patient-specific mitral valve velocities acquired from echocardiography and pressure/velocity profiles at the pulmonary vein inlets would improve the simulations (50).

## 6. Conclusions

LAA blood stasis risk, as quantified by LAA *t_m_* and *C*_∞_, is significantly affected by the mean value of inlet flow (i.e., cardiac output), but not by temporal pattern of the inlet flow. In addition, subject-specific hematocrit is also an important factor and should be considered as one of the input variables for the CFD simulations. Therefore, the subject-specific LAA blood stasis risk can be reliably estimated using subject-specific LA and LAA 3D geometries, subject-specific hematocrit, and subject-specific cardiac output, without any need for subject-specific PV blood flow waveform. Further, at least 20,000 s of tracer concentration transport simulation is needed to calculate LAA *t_m_* reliably and consistently. These results will be used to adjust our CFD-based simulation methodology for calculating LAA *t_m_* and *C*_∞_ in future stroke risk stratification studies.

## Data Availability

The source codes presented in this study can be found in online repositories. The names of the repository/repositories and accession number(s) can be found below: https://github.com/sorooshsanatkhani/LAA-AF-Stroke.

## Footnotes

^1^Vectors and other variables are presented in bold and italics fonts, respectively.
