# Supplementary material for: Subject-specific factors affecting particle residence time distribution of left atrial appendage in atrial fibrillation: A computational model-based study
Source: Front Cardiovasc Med. 2023 Mar 13;10:1070498. doi: 10.3389/fcvm.2023.1070498 (PMC10040531; doi:10.3389/fcvm.2023.1070498)
Supplement: Supplementary file 1 [file Datasheet1.pdf]

SUPPLEMENT

In this study, multiple linear regression was utilized to examine the relationship between various indices (such as cardiac output, pulsatility, hematocrit, etc.) and mean residence time, t_m, and asymptotic concentration, C_∞. Four models were designed in total, 2 presented in Equation (6) and 2 in Equation (8). Data presented in **Table S1** indicate that there are no correlations between independent predictor variables in Equation (6). The Iteratively Reweighted Least Squares (IRLS) method was employed (51) in all multiple linear regression estimations. **Figure S1** demonstrates randomness of the residuals and that they are almost homoscedastic.

TABLE S1 The correlation matrix of independent variables in Equation (6)

CO: cardiac output; Sys_p: normalized systolic peak; Rev_p: normalized reversal peak.

	CO	Sys_p	Rev_p
CO	1	< 0.01	-0.06
Sys_p	< 0.01	1	-0.06
Rev_p	-0.06	-0.06	1

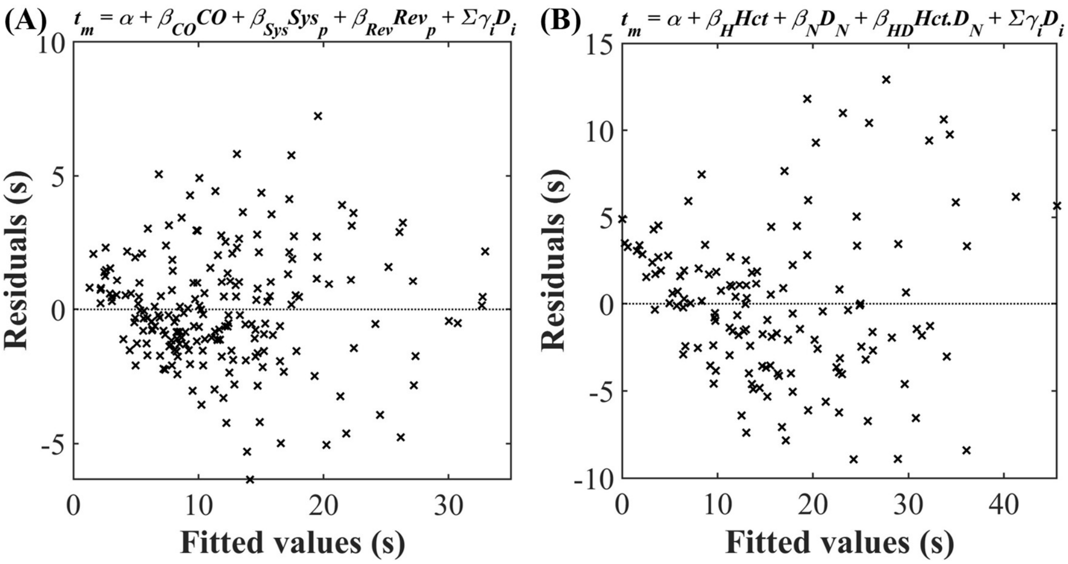

FIGURE S1 Residuals of regression models for mean residence time

(A) Residual plot of the regression model for mean residence time, t_m where infow parameters were used as independent variables. (B) Residual plot of the regression model for t_m where the effect of Hematocrit, rheology model, and their interactions were tested. *CO*: cardiac output; Sys_p: normalized systolic peak; Rev_p: normalized reversal peak.
